# The right people, in the right place—assessing the impact of a new outreach model for paediatric neurology specialist services in Myanmar

**DOI:** 10.3389/frhs.2026.1669010

**Published:** 2026-02-05

**Authors:** Kyaw Linn, Haymar Han, Aye Mya Min Aye, Chaw Su Hlaing, Ayemu Saan, Khine Mi Mi Ko, Marcus Wootton

**Affiliations:** 1 Department of Paediatric Neurology, Yangon Children’s Hospital, Yangon, Myanmar; 2 Childrens Nursing, London South Bank University, London, United Kingdom; 3 International Academy, Royal College of Nursing, London, United Kingdom

**Keywords:** access to specialist care, cost-effectiveness, health workforce shortages, low- and middle-income countries (LMIC), Myanmar (Burma), outreach healthcare services, paediatric neurology, telemedicine

## Abstract

**Background:**

Myanmar has a longstanding and severe shortage of paediatric neurologists, with only 11 specialists serving an estimated 14 million children, most of whom live in rural areas with limited access to tertiary care. This workforce constraint, combined with high out-of-pocket costs and long travel distances, creates substantial barriers to timely diagnosis, treatment, and follow-up for children with neurological conditions. In response to the growing burden of childhood neurological disorders and persistent inequities in access to specialist services, a blended outreach and telemedicine model was developed to extend paediatric neurology care to underserved regions beyond major urban centres.

**Methods:**

A hub-and-spoke model was implemented, linking paediatric neurologists at Yangon Children's Hospital with general paediatricians in seven regional public hospitals. The model combined quarterly in-person outreach clinics by paediatric neurologists from Yangon supplemented with ongoing virtual support delivered through telemedicine and mobile messaging to support continuity of care. Routinely collected data on clinic activity, diagnoses, and costs from 2017 to early 2020 were analysed to assess service reach, impact, and cost.

**Results:**

Between 2017 and 2020, the programme facilitated 2,603 patient consultations. Epilepsy was the most common diagnosis (54%), followed by cerebral palsy (12%). The blended model enabled more efficient use of limited specialist time, with pre-clinic coordination improving case triage and care consistency. Cost analysis demonstrated substantial reductions in patient-related costs, ranging from 81% to 98% per hospital. The mean cost per patient consultation decreased from US$193 under the standard tertiary referral model to US$7 under the outreach model. The programme also supported local capacity building through continuing medical education, strengthened referral pathways, and enhanced regional clinical networks.

**Conclusions:**

This evaluation demonstrates that a hybrid outreach and telemedicine model can deliver accessible, high-quality, and cost-effective paediatric neurology services in a low-resource setting. By leveraging existing national infrastructure and integrating local providers, the model improved access to specialist care, reduced financial barriers for families, and contributed to the long-term capacity of the system. The approach offers a scalable framework for other specialities and health systems facing similar constraints and supports progress towards universal health coverage.

## Introduction

Before the political crisis of 2021, Myanmar had an improving health picture, with significant reductions in neonatal and paediatric mortality rates (1). However, substantial regional variations in specialist expertise and care available to children remained (2). Many children in Myanmar's rural communities, 70% of the total population, live in poverty (3) and experience social deprivation, conditions that both drive neurological morbidity and mortality whilst limiting access to effective treatment.

Paediatric neurological conditions in Myanmar represent a substantial yet largely under-recognised burden within an already fragile health system. For example, evidence from population-based studies demonstrates a wide treatment gap for childhood epilepsy, with many children remaining undiagnosed, untreated, or managed with inappropriate regimens (4).

At the same time, preventable neonatal infections such as sepsis and meningitis continue to occur at high rates in both urban hospitals and underserved border regions, contributing significantly to later cerebral palsy, epilepsy, and broader neurodevelopmental impairment (5, 6).

However, evidence across several LMICs shows that neurological conditions consistently receive less political and financial priority than infectious diseases or malnutrition (7, 8). The negative impact of this lack of focus on the broader population's health and long-term outcomes is highly significant.

Due to a lack of specialist care in Myanmar, neurodevelopmental disorders are commonly identified late due to limited screening, scarce specialist services, and minimal access to speech, occupational, and behavioural therapies (6).

These factors combine to place Myanmar's children at greater risk of a neurological condition from birth and then disadvantage them at every point of potential successful intervention and treatment.

Paediatric neurology is increasingly recognised as a critical speciality in low-resource settings, where declining infant mortality rates and expanding paediatric populations contribute to a growing demand for neurological services. This trend is particularly evident in low- and middle-income countries (LMICs), including Myanmar, which is experiencing some of the most pronounced increases in need (9) as its young populations grow.

Myanmar's challenges in health workforce development mirror wider global inequality in access to specialist care; more than 70% of countries classified as low-income by the World Bank (10) lack consultant-led paediatric neurology services in all areas (11). Myanmar faces substantial challenges in establishing and maintaining adequate paediatric neurology services. At the time of this service evaluation, there were only 11 paediatric neurologists across the country serving a paediatric population of about 14 million children (26% of the total population) (1).

Telemedicine has a long history, dating back to the mid-twentieth century, with the widespread adoption of shortwave radio (12). The delivery model has remained essentially unchanged in most instances, with health workers interacting with patients via a communication platform. The capacity to supplement these consultations with specialist assessment through physical clinical examination is often absent, and medical input is remote via video link.

This traditional model of telemedicine has been positively evaluated (13, 14) but is limited in more specialist care owing to the lack of “hands-on” specialist clinical assessment at the patient's end of the interaction. More broadly, there is evidence of fragmented care in the periods between activities (15).

The positive impact of high-quality specialist paediatric neurological assessment and treatment is well evidenced with significant improvements in development noted, particularly when interventions start before the age of two (16).

Given Myanmar's constrained workforce, political instability, and limited rehabilitation infrastructure, the cumulative effect is a growing population of children living with preventable or manageable disability. Addressing paediatric neurological conditions is therefore not only a clinical imperative but also a broader social and economic necessity, as early identification and intervention are critical determinants of long-term functioning, educational participation, and family wellbeing.

The model described in this paper blended a quarterly specialist mobile clinic with telemedicine observation in the interim. Unlike classical outreach models, which rely on intermittent visits with limited continuity, this model integrates regular in-person specialist assessment with structured telemedicine follow-up between visits.

The model eliminated the need for children to travel to a tertiary centre (as had previously been the practice) and instead used face-to-face consultations with a specialist neurologist at the child's local hospital.

Follow-up and support were provided by telemedicine adapted from the classic model, founded on the concept of doctor-to-patient interaction mediated through an online platform, to one which added a non-specialist doctor who would undertake a physical assessment of the patient under instruction from the specialist, in this case, a paediatric neurologist, who provided clinical input virtually.

Despite growing recognition of the burden of childhood neurological conditions in low- and middle-income countries, there remains a marked absence of evaluated service-delivery models that decentralise paediatric neurology care beyond tertiary centres. Existing literature from LMICs broadly describes either conventional referral-based systems or fully virtual telemedicine approaches, both of which are limited in settings where specialist numbers are extremely low and reliable access to diagnostics and rehabilitation is constrained.

There is little published evidence examining hybrid models that combine periodic in-person specialist outreach with structured, ongoing telemedicine support to regional hospitals. As a result, policymakers and health system planners lack practical evidence on how specialist paediatric neurology services can be extended equitably, sustainably and at scale within resource-constrained settings.

This service evaluation recognises the burden and barriers to specialist services by examining the implementation, activity profile and costs of a blended outreach–telemedicine model designed to expand access to paediatric neurology services across regional hospitals in Myanmar.

We describe a new approach through a model of blended outreach clinics and telemedicine.

This evaluation aimed to:

Describe the activity and diagnostic patterns of the outreach clinics
Assess the extent to which the blended model improved access to paediatric neurology services in regional hospitals.
Compare the direct out-of-pocket costs between the outreach model and the traditional referral pathway.

This paper presents the impact and estimates the per-patient cost of an innovative blended approach to increase the reach of specialist neurology services for children in Myanmar.

Given the clear challenges in accessing paediatric neurology services across the country, a blended outreach model offers a practical way to strengthen regional care while reducing the need for long-distance travel.

## Materials and methods

### Study design

This was designed as a retrospective observational service evaluation of an existing outreach programme, using routinely collected clinical and administrative data from participating hospitals to describe activity, diagnostic patterns and costs associated with the blended paediatric neurology outreach model over the implementation period.

This study assessed the scope impact and cost comparison (compared to the “best case” pre-existing system) of using outreach paediatric neurology specialist services, enabled through a “hub and spoke” model of support to regional hospitals across Myanmar, with paediatric neurological expertise provided by the team at Yangon Children's Hospital. All hospitals enrolled in this study were “public” government hospitals, under the direction of Myanmar's Ministry of Health and Sports.

### Programme origin and rationale

The outreach clinic programme was developed in response to a survey of hospital data in 2015 and 2016, which showed that only a third of children with neurological problems (both outpatients and inpatients) treated at the paediatric neurology unit of Yangon Children's Hospital came from outside Yangon. This highlighted a potentially significant unmet need in rural and semi-rural areas.

### Description of the intervention (blended model)

The outreach clinics, where paediatric neurology specialists supported general paediatricians in regional hospitals, were enabled through monthly virtual consultations and quarterly “in-person visits” by three paediatric neurologists from Yangon, who attended regional hospitals from 2017 to early 2020.

Whilst most children were seen on an outpatient basis, the visiting specialist also saw admitted children with neurological problems during outreach clinic visits, providing expert neurological opinion. Continued medical education activities were also conducted for local doctors and nurses.

The high clinical demand and limited specialist workforce mandated that the bulk of support be delivered virtually. The monthly telemedicine clinic was supplemented by “quick question” mobile support from the paediatric neurology team in Yangon via a mobile messaging app group chat.

The paediatric neurology team ran pilot visits to four regional hospitals in 2017 and 2018 (929 face-to-face patient consultations) before rolling out a larger programme in 2019 and 2020 (1,674 face-to-face patient consultations).

### Patient inclusion, case definitions, and data sources

Data, including patient consultations and diagnoses, were manually extracted and analysed by clinicians to assess the programme's impact. Weaknesses in routine hospital data systems meant it was not possible to distinguish reliably between new and returning patients; therefore, the term “patient consultation” is used throughout as a combined category encompassing both first and follow-up visits.

While follow-up consultations in paediatric neurology can be clinically complex, the inability to distinguish visit type reflects limitations of routine data systems and reinforces the need for improved clinical documentation in future evaluations.

All children assessed by the paediatric neurology team during outreach visits, as well as those reviewed as inpatients at participating regional hospitals, were included in the evaluation. Because clinical documentation did not consistently differentiate between inpatient and outpatient encounters, both were analysed using the same diagnostic recording process.

A “case” was defined as any consultation in which a neurological diagnosis was made or confirmed by the visiting paediatric neurologist or by a regional paediatrician under their direct in-person supervision. No additional inclusion or exclusion criteria were applied, allowing the evaluation to reflect the full spectrum of neurological presentations managed through the outreach programme.

### Data extraction and reliability

A standardised data collection template was used to promote consistency across sites, capturing variables including primary neurological diagnosis, hospital, and date of consultation. No individual-identifying information was collected, and the information required for the cost comparison was collected at the hospital level.

Due to workforce constraints and variability in the record systems, it was not feasible to conduct a second review or an independent verification of the extracted records. This represents a limitation of the evaluation and may have introduced minor inconsistencies or transcription errors. However, every effort was made to ensure accurate and complete data capture within the constraints of the setting.

For our evaluation, we excluded “quick question” via messaging app as an outcome measure because it was not comprehensive, and virtual consultations were not included in the consultation numbers used to manage the existing caseload, rather than to assess new patients. We have chosen to include them in the service information to provide a complete description of all programme elements.

For clarity, virtual consultations and “quick question” messaging exchanges were part of the overall service but were not included in the quantitative dataset used for diagnostic summaries or cost analysis. Only face-to-face consultations during in-person outreach visits were used for numerical analysis.

### Cost analysis

Given the clear evidence that out-of-pocket expenses are a significant barrier to successful referral from regional to tertiary care (17, 18), we compared the costs of the outreach model with those of standard referral to Yangon. This comparison assessed the direct expenses associated with a paediatric neurologist travelling to regional hospitals—public transport, basic accommodation and food—vs. the equivalent costs children and families would incur when travelling to the tertiary centre. Staff salaries were excluded, and the analysis represents a “best case” scenario, assuming all referred patients were able to make the journey. We recognise the reality of widespread evidence that financial hardship frequently prevents families in low-income settings from accessing specialist services in Myanmar (19).

The analysis focused on direct, out-of-pocket costs (transport, accommodation, food), which constitute the most immediate financial barriers to specialist access in Myanmar. The same cost categories were applied to both models to enable a fair comparison. Indirect costs—such as lost caregiver wages, children missed school days and longer-term socioeconomic impacts of delayed neurological care—were not included due to limitations in data (Table 1).

**Table 1:** Cost comparison for equivalent specialist coverage, comparing the cost of patient travel to the tertiary centre to the price of a mobile clinic.

Hospital	Standard referral model	New outreach model
	Average cost per patient and carer attending a face-to-face consultation at the tertiary centre (95% CI)	Cost per patient consultation of a paediatric neurologist visit at each regional hospital (3-day visit)
Kale Hospital	$238	$4
Magway Hospital	$9	$2
Kengtung Hospital	$441	$29
Bago Hospital	$66	$1
Pathein Hospital	$77	$2
Mawlamyine Hospital	$77	$1
Myitkyina Hospital	$441	$8
Mean Average	$193	$7

Exchange rate of $1 (US dollar) to 1,430 MMK (Myanmar Kyat).

The exclusion of broader economic consequences is notable given the substantial long-term burden of neurological conditions in LMICS (20). Programme development costs and staff salaries were also excluded.

Although a health economist did not formally advise on the costing framework, the approach followed Drummond et al.'s principles for descriptive economic analyses in telemedicine and global health (21), which emphasise specifying the analytic perspective, defining included cost components, and acknowledging data gaps (21).

Accordingly, the findings should be interpreted as conservative and illustrative of immediate financial burdens rather than as a complete economic evaluation and likely underestimate the broader economic benefits of the outreach model.

### Ethical considerations

This programme was based on a retrospective review of clinical data collected as part of routine clinical care, supplemented by cost estimates calculated by the authors.

All patient information was anonymised before analysis, and no identifiable details were used. Identifiable data (such as names and hospital numbers) were viewed solely to locate the correct clinical records and were removed during the extraction process; no identifiable information was entered into the study dataset.

Since no additional clinical procedures were performed and the data were part of usual care, individual consent was not required.

Ethical approval was formally waived by the Institutional Review Board of Yangon Children's Hospital, which confirmed that the evaluation met criteria for a service evaluation rather than human subject's research. This approach followed international, local and institutional guidelines for health service evaluations (22). Telemedicine and mobile messaging used for clinical support were conducted on password-protected devices, and no patient information from these communications was stored or used in the analysis.

## Results

We present the number and diagnosis mix of patient consultations for the four pilot hospitals, which were supported in the pilot phase between 2017 and 2018, and then for the whole programme, which ran from 2019 into early 2020, when the coronavirus pandemic began. This was followed in 2021 by the significant changes in Myanmar's political situation, which fragmented the health system.

### Programme activity and consultation volume over time

**Figure 1:**
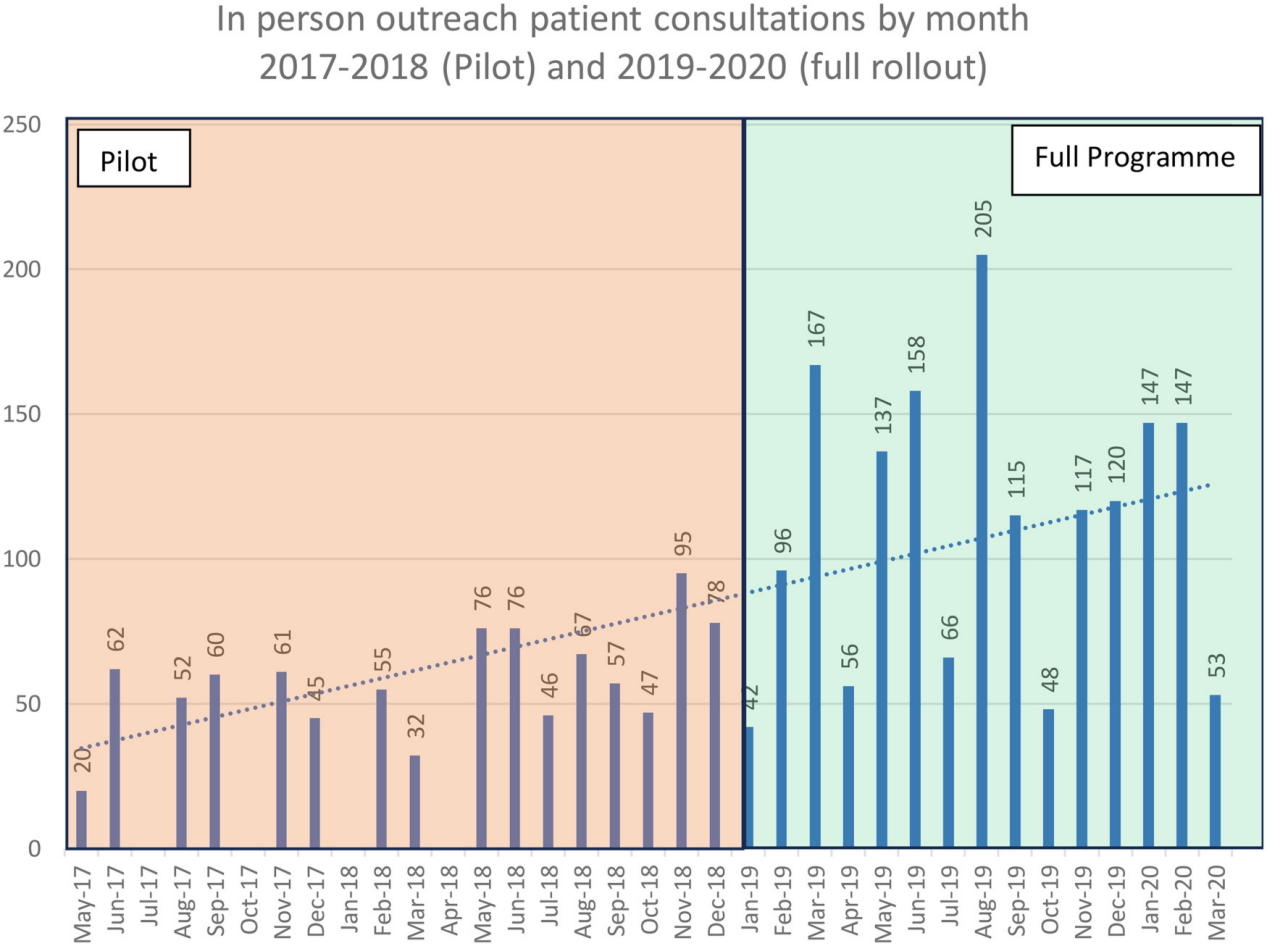
Outreach clinic patient consultations by month across seven regional hospitals, 2017–2020 (pilot in orange and programme in green).

Figure 1 shows a steady rise in month-on-month patient virtual consultations throughout the pilot and into the programme's full rollout. In total, 2,603 virtual consultations were undertaken, with a mean of 58 per month during the pilot and 111 per month during the whole programme. Overall, the programme undertook 83 virtual consultations per month. The variance across months ranged from no activity to 205 consultations, with a clear upward trend throughout the programme.

**Figure 2:**
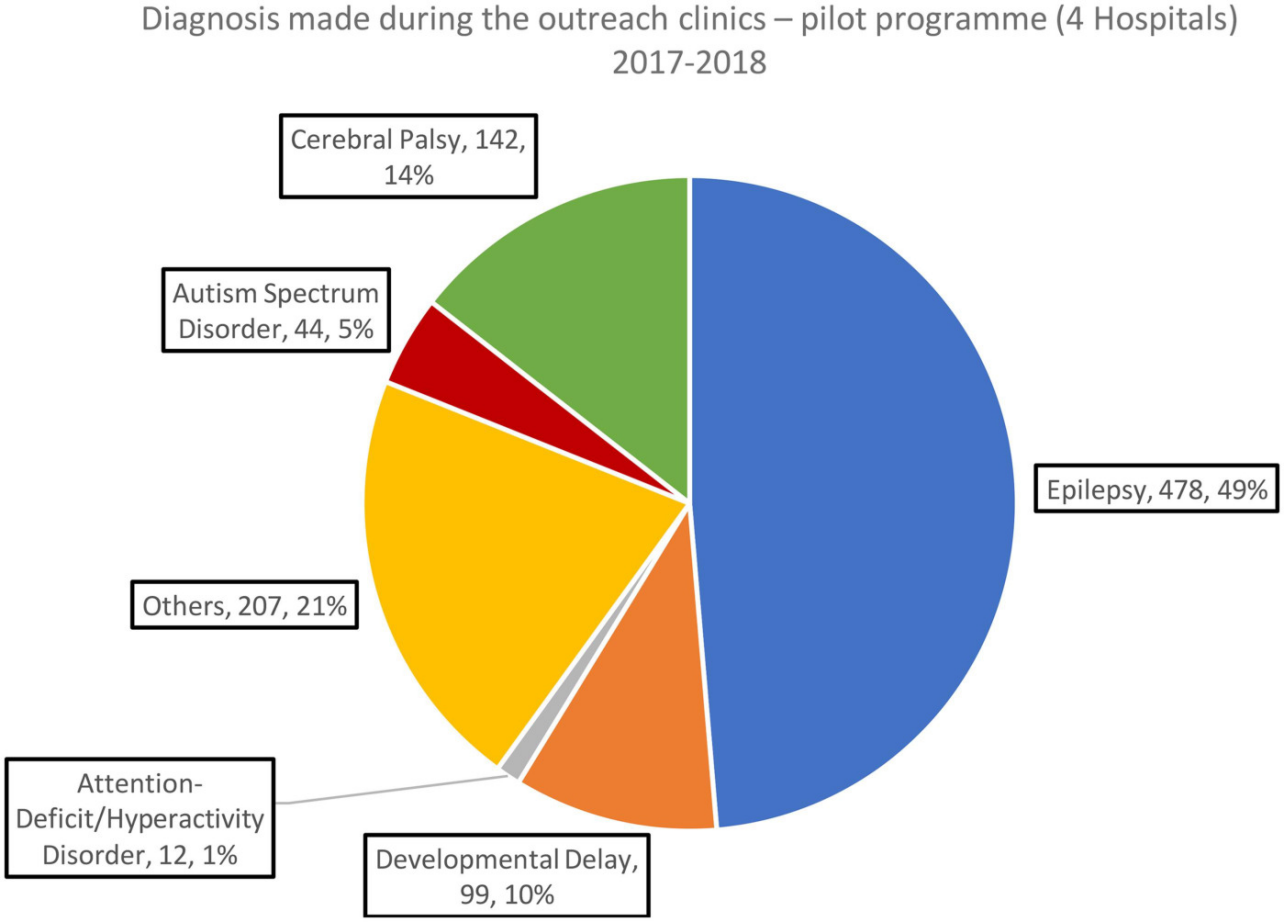
Diagnosis made during the outreach clinics – pilot programme (all patient consultations) 2017–2018.

**Figure 3:**
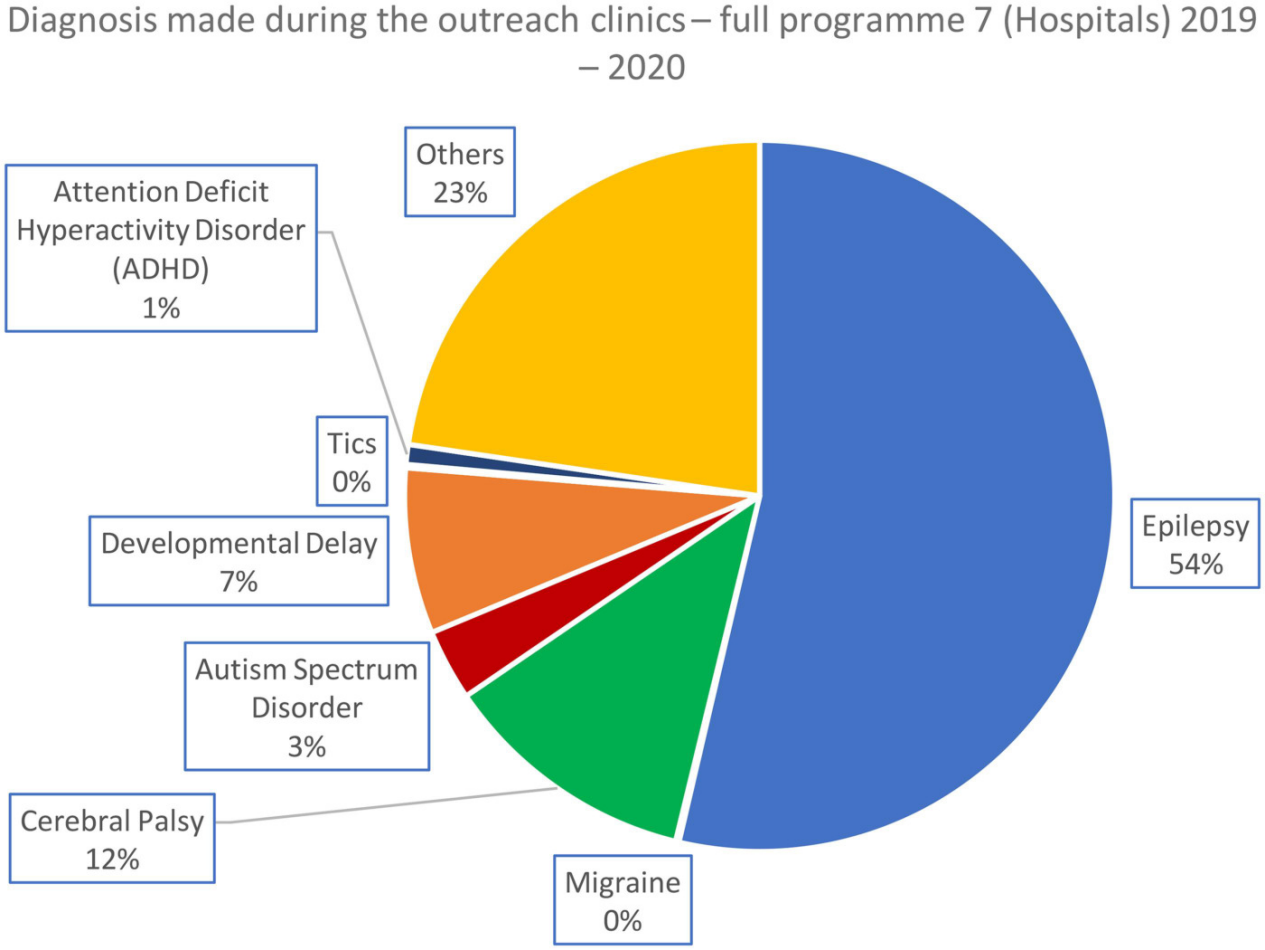
Diagnosis made during the outreach clinics (all patient consultations) – complete programme 2019–2020.

**Figure 4:**
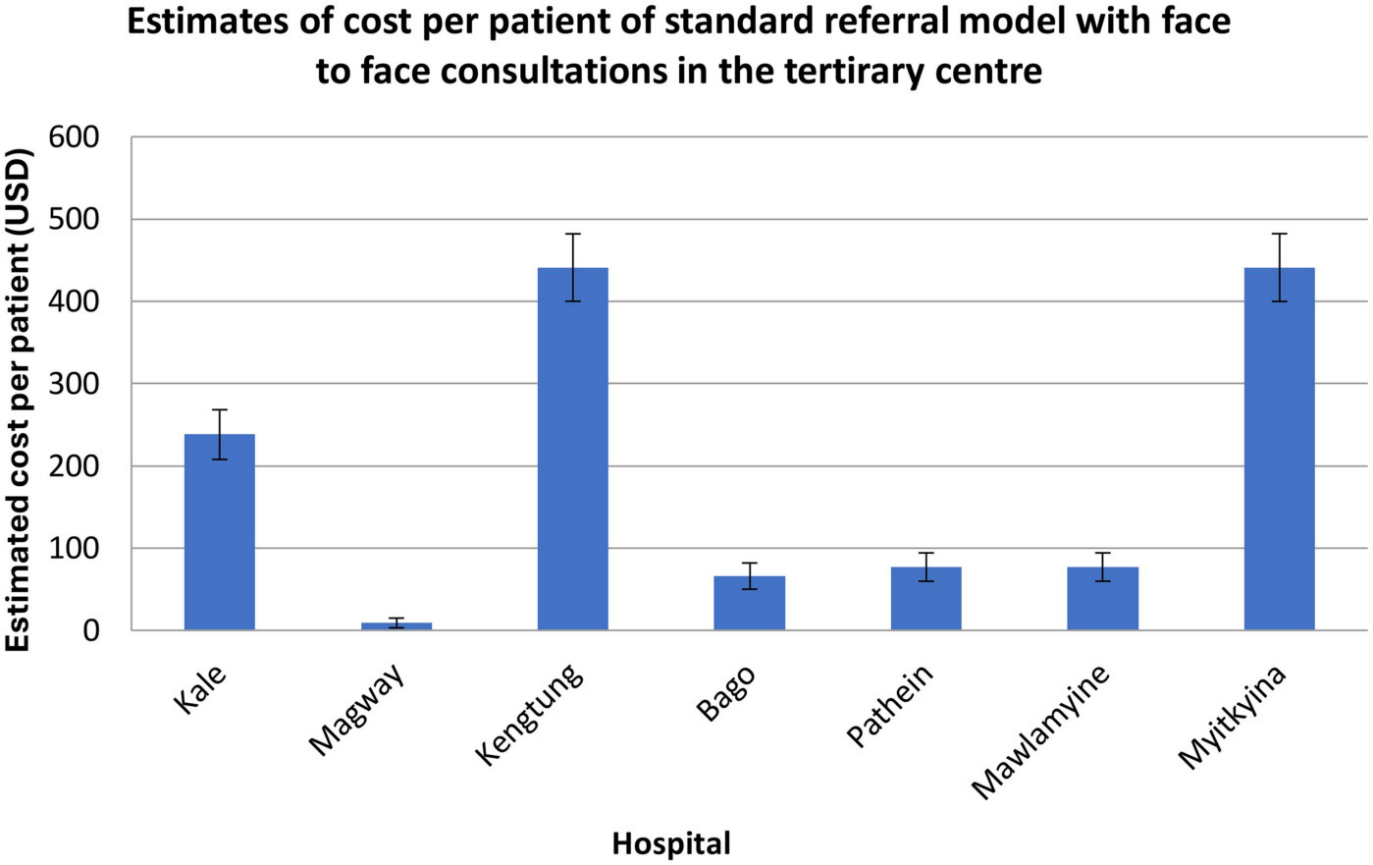
Estimate of cost per patient of the standard model where face-to-face consultations occur in the tertiary centre.

**Figure 5:**
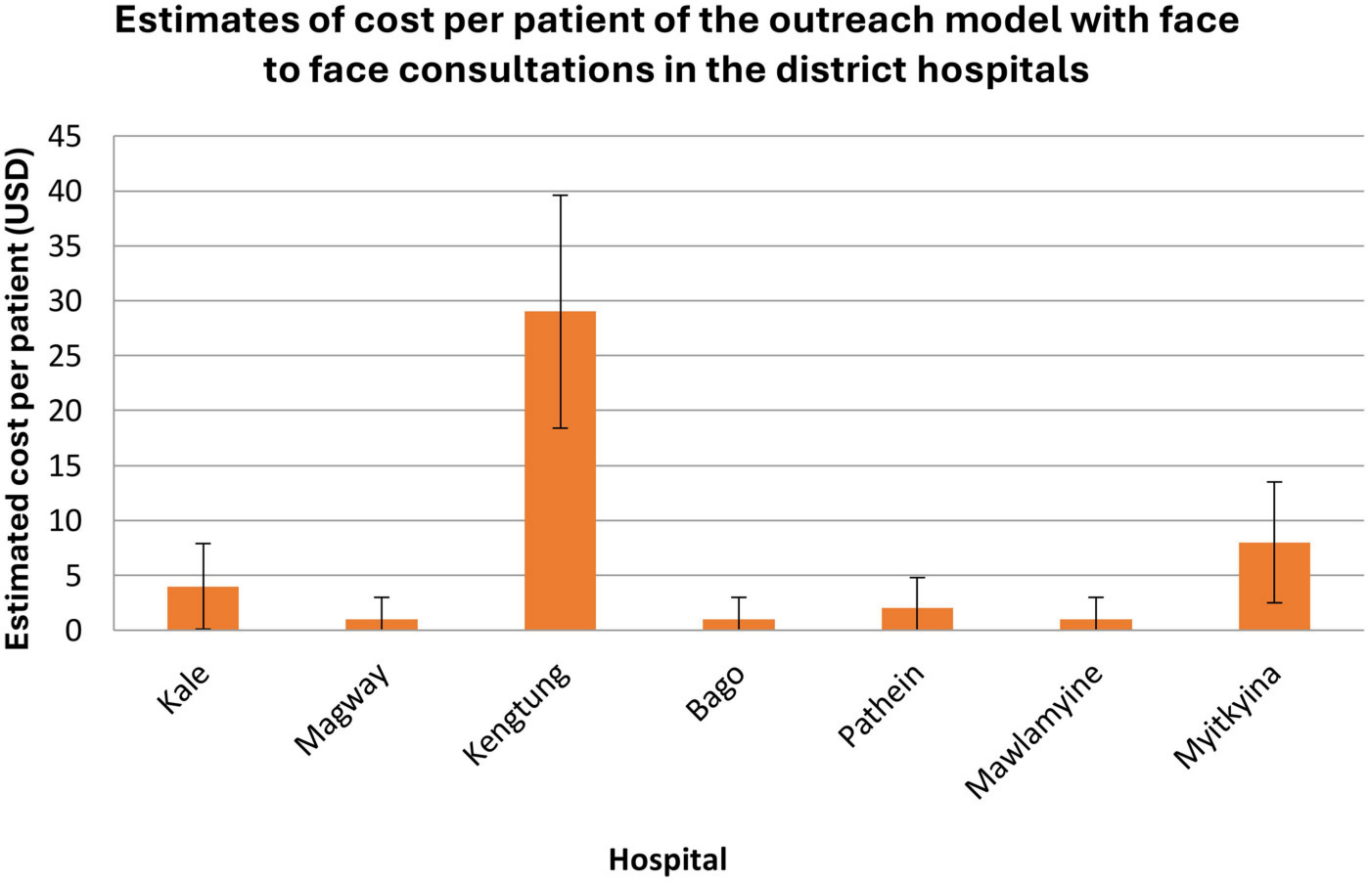
Estimate of cost per patient of the outreach model, where face-to-face consultations occur in the district hospitals.

### Diagnostic profile of outreach consultations

Across both phases of the programme, epilepsy remained the predominant diagnosis, increasing from 49% in the pilot (2017–2018) to 54% in the full rollout (2019–2020). In contrast, the relative proportions of neurodevelopmental conditions—including cerebral palsy, developmental delay, and autism spectrum disorder—fell slightly in the expanded programme. The “Other” category grew modestly (21%–23%), reflecting a broader mix of presentations as services reached more hospitals and a wider catchment population. Overall, the full rollout largely mirrored the pilot's diagnostic pattern but showed greater diagnostic diversity and a shift toward a higher proportion of epilepsy cases.

In the pilot programme, just over half of all diagnoses were epilepsy (Figure 2). In all cases, we present proportions and actual numbers of patient consultations. The diagnostic distribution for the complete programme (2019–2020) is shown in Figure 3.

The costs of facilitating the clinics (Figures 4, 5) were compared with the equivalent costs of patients travelling to a tertiary centre for the same level of specialist assessment and treatment. These costs were calculated by adding the components presented in Table 2. Costs remain the same whether patients attended the clinic as new or follow-up patients.

**Table 2:** Direct and indirect costs.

Cost category	Examples
Direct costs (included in analysis)	
Transport	Bus or taxi fares for a child and caregiver travelling to Yangon (standard model) or for a paediatric neurologist travelling to regional hospitals (outreach model).
Accommodation	One night for a patient and caregiver in the standard model, or four nights for the neurologist during outreach visits.
Food	Daily subsistence allowance (1 USD per day), applied equally to patients/caregivers (standard model) and to the neurologist (outreach model).
Online platform subscription	Annual cost of the video-consultation platform used to support the outreach model.
Indirect Costs (not included in analysis	
Caregiver income loss	Lost wages due to travel or time off work required to attend specialist appointments.
Child's missed school days	Educational disruption resulting from long-distance travel.
Additional transport needs	Local transport within Yangon, or between rural villages and regional hospitals.
Long-term socioeconomic impact	Costs associated with delayed diagnosis, disability progression, or reduced family productivity.
Programme/staff costs	Salaries of specialists, administrative staff time, or programme development/support.

### Cost comparison between the standard referral model and the new outreach model

The cost-saving percentage ranged from 81% to 98%, depending on the distance between the regional hospital and Yangon, where the tertiary centre was located.

### Variation in costs by hospital location and distance

Across hospitals, the cost estimates showed substantial variability, reflecting Myanmar's diverse geography and transport infrastructure. Hospitals located furthest from Yangon, such as Myitkyina and Kengtung, generated the greatest savings because patient travel typically involved multi-day road journeys with high accommodation and food costs. In contrast, hospitals closer to Yangon, such as Bago and Mawlamyine, still demonstrated considerable proportional savings despite lower baseline travel costs.

These differences highlight the sensitivity of out-of-pocket costs to distance and travel time and support the argument that outreach models disproportionately benefit families living in remote areas.

These cost differences also reflect the broader issue of geographic inequality in Myanmar. Families living far from Yangon face much higher travel costs to access specialist care, meaning the outreach model provides particular benefit to children in the most remote regions.

The uncertainty intervals presented for the standard model reflect natural variation in ticket prices, fuel costs and seasonal fluctuations in accommodation fees. While these were not calculated through formal probabilistic modelling, they offer a realistic range of expected expenditure for families undertaking travel to Yangon. In contrast, outreach model costs varied only minimally across hospitals because they relied on predictable accommodation transport costs for the visiting neurologist.

It is important to emphasise that these cost comparisons are descriptive rather than inferential. The analysis does not test statistical differences between models nor estimate population-level economic impact. Instead, the figures illustrate the magnitude of financial relief delivered by decentralising specialist neurology care. Even under conservative assumptions, reductions of 80%–98% demonstrate the considerable economic advantage of providing services locally rather than relying on long-distance referral pathways.

### Additional observations from programme implementation

In addition to scheduled outpatient consultations, visiting specialists provided opportunistic neurological reviews for hospitalised children during outreach visits. Towards the later stages of the programme, some regional hospitals began pre-screening cases before outreach visits, allowing specialists to focus on children with the greatest clinical need. In contrast, others were redirected to more appropriate services such as physiotherapy or ophthalmology.

These observations were not included in the quantitative analysis but provide important contextual insight into how the outreach model evolved in practice.

## Discussion

This evaluation highlights the successful implementation of Myanmar's first mobile specialist outreach programme, providing paediatric neurology care in regional hospitals. It demonstrates a scalable and cost-effective model of care that bridges the gap between limited specialist availability and significant unmet patient needs, particularly in rural areas. This evaluation shows the feasibility and impact of a hybrid outreach and telemedicine model for paediatric neurology in Myanmar, a country facing significant disparities in access to specialist healthcare. By integrating periodic in-person clinics with continuous virtual support, the initiative effectively extended specialist services to underserved rural populations, a vital component in achieving universal health coverage.

### Key findings and interpretation

#### A viable model

The blended approach of in-person outreach supplemented by telemedicine follow-up via mobile platforms proved particularly well-suited to Myanmar's needs. Neurology is a highly specialised field, and training sufficient numbers of clinicians to provide a more traditional in-person service could take decades. Provide a practical, intermediate solution that uses the country's existing capacity rather than relying on inconsistent international support.

This programme evaluation demonstrated a viable hybrid delivery model, with a steady, incremental rise in clinic attendance throughout its duration. The programme facilitated 2,603 specialist consultations for children across seven regional hospitals, with epilepsy (54%) and cerebral palsy (12%) being the most prevalent conditions.

The model's design—combining scheduled visits with telemedicine follow-ups—enabled consistent care delivery despite limited specialist availability. Pre-clinic coordination allowed local hospitals to consolidate cases, optimising the use of specialist time and resources.

This hybrid approach to providing specialist paediatric neurology has been successfully evaluated, including in Canada (23) and the USA (24). It should, however, be noted that these interventions were conducted during COVID-19 and did not incorporate face-to-face assessment. They were also conducted in high-resource settings. We are not aware of a comparable model being undertaken before in Paediatric Neurology in a low-resource setting. The successful implementation of this programme signals the viability in this setting.

### Widening specialist reach

Childhood neurological disorders in low-resource settings commonly present with epilepsy (24) and cerebral palsy (25) as the predominant diagnoses, which aligns with our findings. The steady rise in patient consultations across the programme is consistent with evidence that decentralised services often reveal significant unmet need that is not apparent in tertiary-level hospital data (26).

This approach allows tertiary centre specialists to extend their reach without relocating and equips regional doctors with the knowledge and confidence to manage common neurological conditions or refer appropriately. This type of model is particularly applicable to specialities such as paediatric neurology, which require ongoing follow-up and specialist care throughout childhood.

### Cost-effectiveness and accessibility

Financial analysis revealed that the outreach clinics reduced costs per patient consultation by an average of 7 USD per visit, compared to 193 USD per consultation with traditional referrals to tertiary centres. These costs were primarily borne by the Ministry of Health and Sports, which funded specialist travel. As a result, the financial burden was almost entirely removed from patients and their families, who only needed to travel to their local hospital rather than to a tertiary centre, sometimes hundreds of miles away. This substantial saving is particularly significant in contexts where healthcare expenses often deter families from seeking necessary care (27).

Similar economic evaluations in LMIC settings have emphasised costs to families caring for a child with a lifelong diagnosis and the impact on wider areas of life, including school attendance (28). The models we present eliminates the need for travel, thereby positively impacting school attendance. Broader economic analyses also show that chronic and long-term conditions impose a substantial financial burden on households, increasing the risk of catastrophic expenditures.

Childhood disability, including neurological conditions, is associated with particularly high lifetime economic costs for families and society, underscoring the importance of accessible, decentralised services (29). The outreach model we enacted directly addresses these challenges by eliminating the need for long-distance travel.

The decision not to include staff salaries in the cost comparison aligns with established approaches for early-stage service evaluations, where fixed system costs remain unchanged between models of care and are therefore excluded (30) While more detailed modelling would require long-term outcomes and patient-level cost data, the descriptive comparison used here reflects recommended methods for assessing new service-delivery models in low-resource health systems (21).

These findings are also relevant beyond Myanmar. Many low- and middle-income countries face similar challenges, including long travel distances, limited specialist distribution, and high out-of-pocket costs. This suggests that the blended outreach model may be applicable in other LMIC settings with comparable health system constraints.

The model not only alleviated direct financial burdens on patients but also minimised travel-related hardships, including disruptions to parental work and school attendance, thereby enhancing overall accessibility.

The financial impact of the outreach model aligns with broader evidence from LMIC settings, which shows that travel-related expenses are among the most significant barriers to accessing specialist care. For chronic neurological conditions, which often require repeated follow-up over many years, eliminating the need for long-distance travel is particularly important. Research from similar contexts indicates that a single journey to a tertiary centre can represent several weeks of household income and may force families to defer or abandon care altogether. The shift to local specialist access, therefore, has important implications for equity and care continuity.

The exclusion of staff salaries from the cost comparison warrants clarification. Salaries for clinicians and administrative staff would be incurred under both models of care and therefore do not influence the comparative difference between the standard and outreach pathways. This approach follows accepted practice for descriptive economic evaluations and was taken to focus on the variable, out-of-pocket costs, most relevant to families. However, omitting workforce costs means the analysis does not constitute a complete health system evaluation, and future studies will need to consider the broader resource implications of programme scale-up.

Despite these limitations, the analysis provides a clear indication of the extent to which decentralised specialist services can reduce financial barriers for families. The cost savings described here are likely conservative, given the additional indirect costs avoided by not travelling—such as lost income, disruption to schooling, and the longer-term consequences of delayed diagnosis.

### Local capacity building and system strengthening

Beyond direct patient care, the programme contributed to health system strengthening. Notably, the neurology clinics served as a platform for continuing medical education (CME) and hands-on learning for local healthcare providers, contributing to longer-term capacity-building. The impact of this lies outside the scope of this evaluation, but further research on the secondary effects of this type of model would be valuable.

While the majority of children were managed on an outpatient basis, the outreach clinics also enabled opportunistic expert reviews for inpatients, offering timely specialist opinions for acutely unwell children. Learning was bidirectional, as specialists were provided with a window into the realities of paediatric care outside a large city like Yangon, where overall child health outcomes are better than in rural and semi-rural areas of the country (17). Towards the latter stages of the programme, some regional hospitals began pre-screening patients, ensuring that those most in need received specialist attention. In contrast, others were appropriately referred to different services such as ophthalmology or physiotherapy.

The consistent involvement of district clinicians in telemedicine consultations between outreach visits further embedded knowledge and strengthened clinical networks. This model not only expanded access to care but also pointed policymakers toward key areas for improving the system.

### Challenges and adaptations

The initiative faced challenges, including limited access to advanced diagnostics and the need for referrals to other specialities. Alongside this, there were frequent challenges with difficult access to ongoing in-person advanced-level care and some medications. The core group of neurologists in Yangon undertook this work in addition to their ongoing clinical duties, which continued throughout the programme. There is a ceiling of intervention scale at which this model of delivery becomes non-viable. Still, we were unable to identify precisely where this cut-off point was during this evaluation. Telemedicine clinics discussed emergency case management between outreach clinic times; however, clinical input was not comprehensive due to the virtual nature of the clinics.

### Scalability and workforce sustainability

Although the outreach model has demonstrated clear benefits, its long-term expansion is limited by the small number of paediatric neurologists in Myanmar, who undertake this work in addition to their full clinical duties. Sustaining and scaling the service will require strategic support, including training general paediatricians in the management of common neurological conditions and developing structured referral pathways. In the longer term, national workforce planning and collaboration with international partners for training and mentorship may help reduce the burden on specialists and ensure the model can be extended to more regions without compromising quality or clinician wellbeing.

### Broader implementation challenges

In practice, several challenges affected the delivery and consistency of the programme. Access to key medications and diagnostic investigations varied across hospitals, sometimes limiting the care that could be provided. The model also depended on reliable internet access and the ongoing commitment of local clinicians, both of which could fluctuate and impact service delivery. Furthermore, the political instability and broader health system disruptions during this period created real difficulties in maintaining continuity and expanding the programme, underlining the need to plan for service resilience in such settings.

### Strengths of the evaluation

This evaluation has several strengths. It uses real-world data from seven public hospitals, reflecting routine paediatric neurology practice rather than controlled research conditions. The programme had clearly defined components delivered consistently across sites, strengthening internal coherence.

To our knowledge, this is one of the first evaluations of a blended outreach–telemedicine paediatric neurology model in a low-resource setting. These features enhance the relevance and usefulness of the findings for service planning and policy.

## Limitations

This evaluation relied on routine clinical records from government hospitals, and variability in documentation likely affected data completeness and accuracy. It was not possible to distinguish new from returning patients, meaning some conditions may have been unintentionally double-counted, and patient or caregiver perspectives could not be collected. As a retrospective and primarily descriptive study without a control group, the findings cannot establish causal relationships, nor were long-term clinical outcomes, treatment adherence, or functional improvements systematically tracked. Programme continuity and follow-up were further constrained by significant disruptions in Myanmar, including political instability and the COVID-19 pandemic.

The cost analysis also has limitations: estimates were based on administrative data rather than household expenditure surveys, and only direct costs were included, excluding substantial indirect and longer-term economic burdens. No sensitivity analyses were undertaken, and the comparison assumes that all families would have been able to travel to Yangon—an assumption that likely overstates the accessibility of the standard model. These limitations mean the cost figures should be interpreted as indicative rather than precise; however, they reinforce the rationale for decentralised specialist services and highlight areas where further research is needed to assess clinical outcomes, patient experience and long-term sustainability.

## Policy implications and future directions

This model demonstrates a scalable, sustainable approach to delivering specialist care in low-resource settings by building on existing national capacity rather than relying on external support. Its alignment with global health priorities and its early adoption by other specialities in Myanmar indicate strong potential for broader application across the health system. In a context where transport barriers remain significant, but digital connectivity is increasingly widespread, the success of paediatric neurology outreach offers a compelling case for similar decentralised approaches in other areas of specialist care. While further evaluation of long-term outcomes and system-level integration is needed, the programme already shows considerable value. Beyond the substantial cost savings for families, earlier access to specialist assessment may also reduce avoidable hospitalisations, disability and long-term socioeconomic burden.

Given that many LMICs face similar barriers—including long travel distances, limited specialist distribution and high out-of-pocket costs—the model described here may be adaptable to other low-resource settings.

Several policy actions could support the model's sustainability and expansion. Integrating outreach neurology services into national health system planning would provide predictable funding and embed the approach within routine service delivery. Developing national clinical pathways and clear referral criteria for common childhood neurological conditions would strengthen district-level management and ensure efficient use of specialist expertise. Continued investment in telemedicine capacity and ongoing training for district clinicians would enhance the quality and consistency of remote care and contribute to a more structured, tiered neurological service. Finally, workforce sustainability remains critical, requiring targeted training for general paediatricians, support for new paediatric neurology trainees and strengthened mentorship partnerships. Institutionalising these components would help ensure the model's long-term viability and facilitate its extension to additional specialities and regions.

Future programme development could also include simple electronic data systems or EHR-integrated documentation to improve monitoring, follow-up and evaluation.

This model demonstrates a scalable, sustainable approach to delivering specialist care in low-resource settings by leveraging existing national capacity. Its alignment with global health priorities and early adoption by other specialities in Myanmar indicates strong potential for broader application.

Several policy actions could support sustainability, including integrating outreach neurology services into national health planning, developing standardised referral pathways and strengthening telemedicine capacity.

## Conclusion

This evaluation demonstrates that a blended model combining in-person specialist outreach with telemedicine support can improve access to paediatric neurology services in regional hospitals across Myanmar. The model provided substantial cost savings for families, reduced the need for long-distance travel and offered a practical solution to workforce constraints within the country.

The outreach clinics enabled a significant number of children to receive timely specialist assessment and contributed to improved collaboration between tertiary and regional paediatric teams. While challenges remain—particularly around workforce capacity, diagnostic resources and wider political instability—the evaluation shows that innovative approaches can help bridge existing gaps in specialist care.

These findings offer early evidence that decentralised specialist services, when adapted to local health system realities, can strengthen access and equity for children with neurological conditions.

As the programme evolves, integrating simple digital data tools and supporting ongoing training for regional clinicians may help sustain and expand the benefits demonstrated in this evaluation.

Overall, this blended outreach model represents a feasible and context-appropriate approach for expanding paediatric neurology services in low-resource settings.

## Data Availability

The data analyzed in this study is subject to the following licenses/restrictions: These were hospital records collected by the previous Myanmar Ministry of Health and Sports. They have now been deposed by a military junta. Requests to access these datasets should be directed to woottonm@lsbu.ac.uk.
